# Does access to care affect outcomes of appendicitis in children? - a population-based cohort study

**DOI:** 10.1186/1472-6963-10-250

**Published:** 2010-08-25

**Authors:** Teresa To, Jacob C Langer

**Affiliations:** 1 Child Health Evaluative Sciences, Research Institute, The Hospital for Sick Children, Toronto, Ontario, Canada; 2 The Dalla Lana School of Public Health, Department of Health Policy, Management and Evaulation, Department of Pediatrics, University of Toronto, Ontario, Canada; 3 The Institute for Clinical Evaluative Sciences, Toronto, Ontario, Canada; 4 Department of Surgery, The Hospital for Sick Children, Toronto, Ontario, Canada

## Abstract

**Background:**

The annual number of pediatric appendectomies in Ontario was stable throughout the study period, but with a significant level of regional variations across regions. The objective of this study is to use population-based data to measure the associations and to explain the variations of appendectomy rates with population socio-demographic indicators.

**Methods:**

Appendectomy rates in children aged less than 19 years were calculated from Ontario hospital discharge data from 1993 to 2000. Small area variations in appendectomy and correlations between socio-demographic indicators were studied. Multiple logistic regression was used to measure the risk of negative appendectomy and perforation while adjusting for socio-demographic factors.

**Results:**

The rate of positive primary appendectomy has been stable since 1993 with an average rate of 93.2 per 100,000 children. The negative appendectomy rates showed a significant decline over time from a high of 16.0 in 1994 to 10.2 per 100,000 in 2000 (p < 0.0001). There was a 4-fold regional variation in negative appendectomy with the highest rate of 26.0 per 100,000 in the northern regions of Ontario. After adjusting for socio-economic status, areas of higher percentages of rural living remained a single significant factor associated with a higher chance of negative and perforated appendectomy (OR = 1.28, 95% CI: 1.01, 1.61, p < 0.01 and OR = 1.11, 95% CI: 0.96, 1.28, p = 1.682 respectively). Areas with higher ultrasound use were associated with a lower risk of perforated appendectomy (OR = 0.83, 95% CI: 0.72, 0.95, p < 0.05).

**Conclusion:**

The higher rates of negative and perforated appendectomy in rural populations underpin the influence of access to preventive and primary health care in modifying the odds of appendicitis resulting in surgery.

## Background

Acute appendicitis is a common surgical emergency in children. Recent studies showed that the incidence of acute appendicitis has declined in western countries. The surgical intervention for acute appendicitis has been reported to vary by country, geographic regions, race, sex, seasons, immigrant and socioeconomic status[1-4]. The reasons for this variation are not fully understood. Most epidemiologic studies in appendicitis focused in the role of age, sex, hereditary and dietary influence on the incidence of appendicitis; few had examined the intricacy of the interplay between population demographics and the access to health care on outcomes in children who underwent appendectomies. The objective of this study is to use population-based data from Ontario, Canada to measure the associations and to explain the variations of appendectomy rates with population socio-demographic indicators.

## Methods

### Patient Data

Computerized data on hospital discharges from the Canadian Institute for Health Information (CIHI) were used for Ontario for fiscal years 1993 to 2000. All hospitals and community-based health service facilities in Ontario are mandated to submit discharge data to CIHI. A fiscal year runs from April 1 to March 31 of the following year.

### Socio-Demographic Indicators

Household income, education and ever landed immigrant status were obtained from the 1996 Canada Census population data. The definition of low education is the one used by Statistics Canada. It is defined as the percentage of population 15 years of age and over who had less than a grade nine education. For annual family income, a cut-off at $35,000 or below is used for comparison, a definition of an average family income according to Statistics Canada. Rural and small town (RST) areas refer to the population residing outside Census Metropolitan Areas (CMAs) and Census Agglomerations (CAs). A CMA has an urban core of 100,000 or more and a CA has an urban core of 10,000 to 99,999 residents. CMAs and CAs include all neighboring municipalities where 50 percent or more of the workforce commutes into the urban core. Thus, RST areas represent the non-CMA and non-CA population[5]. The percent of immigrants in the residing area is defined by the number of persons responded to the census as ever been a landed immigrant of Canada. Since the utilization of ultrasound or CT scans may be influenced by the volume of surgeons (supply) working in the region, we used the Canadian National Physician Database (NPDB) to measure the availability of surgeons. The availability or supply of surgeons was defined as the total number of both paediatric and general surgeons working in each region. The NPDB contains data on socio-demographic characteristics (including specialty and place of work) of all licensed physicians in Canada. Diagnostic and therapeutic procedures such as ultrasound or CT scans performed on outpatients in hospitals and in physicians' offices and laboratories are billed to the Ontario Health Insurance Plan (OHIP) database on a fee-for-service basis. In this study, the rate of use of ultrasound was determined by the total number of abdominal ultrasounds billed to OHIP divided by the population of children in the region.

### Inclusions and Exclusions

Children under 19 years of age in Ontario who had a diagnosis of appendicitis with ICD-9 codes 540.0 (perforated), 540.1 (non-perforated) or 540.9 (unqualified) and a surgical procedure code for operations on appendix: appendectomy (ICD-9 code 47.0), or drainage of appendiceal abscess (47.2) in fiscal years 1993 to 2000 were included as having a primary appendectomy[6]. Perforation was only recorded in the presence of positive primary appendectomy where either the diagnosis code was 540.0, or if a drainage procedure of an appendiceal abscess was performed and recorded as a separate procedure. Negative appendectomy was defined as those who underwent appendectomy for a preoperative diagnosis of appendicitis, but did not have appendicitis. Those undergoing incidental appendectomy at the time of an unrelated procedure were excluded from all analyses. Detailed description of this study population is available elsewhere[7].

## Method of Analysis

### Standardization

The method of direct standardization was used in calculating the age- and sex-adjusted rates[8]. The 1996 Canadian census population was used as the standard population in the direct standardization. All rates were calculated per 100,000 children. The 1 degree of freedom chi-square test was used to determine if the rate of an area was statistically different from a standard or referent area[9,10]. Discharge rates were reported by the patient's District Health Council (DHC) area of residence as determined by the residence codes. DHC's are local health planning and advisory organizations that report to the Ontario Ministry of Health and Long-Term Care. A DHC was classified as rural based on the percent of its total population residing in non-CMA or CA regions. Geographic rates were adjusted for age and sex.

### Small Area Variation Analysis

Three commonly used statistics were calculated to measure variation between DHCs in Ontario. The extremal quotient (EQ) is the ratio of the highest to lowest rate. The coefficient of variation (CV), which takes into account the population sizes being worked with, divides the standard deviation of the rates by the average rate and the systematic component of variation (SCV) measures the relative systematic component of variation in rates between regions by subtracting the random component of variance from the total variance[11-13]. These "standard" methods have been widely used by health services researchers in characterizing small area variation[14-17].

### Correlations

Correlations between socio-demographic variables and appendectomy rates were calculated by the Spearman Correlation Coefficients. A p-value of less than 5% was considered statistically significant.

### Regressions

The logistic regression model was used to model the association/risk of high admissions for appendectomies. The outcome variables used were rates of negative appendectomy and perforated appendicitis in each of the 16 DHCs. Independent factors considered in the regression model included socio-demographic variables such as household income, education, percent of the population that was English speaking, percent of the residing area that was defined as rural, the percent distribution of immigrants in the residing areas, the availability of surgeons in the region and the utilization rates of abdominal ultrasound. Univariate regression was used to test the statistical significance of risk factors for appendectomies. Inclusion of covariates in the final multivariable regression model was based in part on patterns of correlation, statistical significance, and evidence of confounding. All analyses were conducted using the SAS statistical package (SAS version 8.0, Cary, North Carolina, USA) was used[18].

## Results

### Trends of Appendectomy

**Figure 1:**
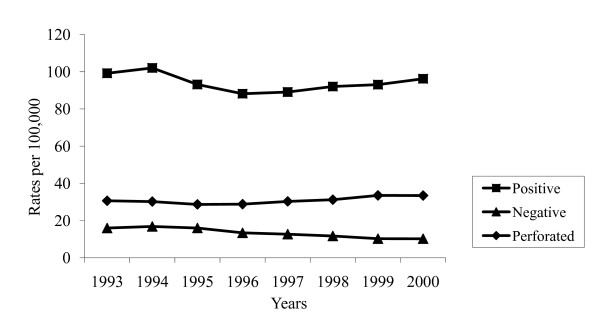
**Rates of positive, negative primary appendectomy and perforated appendicitis in Ontario in children aged under 19 years, 1993 to 2000**.

There were 21,027 positive primary appendectomies in children under 19 years of age in fiscal years 1993 to 2000 in Ontario, previously described by Somme et al.[7] Figure 1 shows the relatively stable positive primary appendectomy rates in Ontario children ranging from 99.2 per 100,000 in 1993 to 96.2 per 100,000 population of children in 2000. Approximately a third of these children were with perforated appendicitis giving a rate of 30.4 per 100,000 in 1993 and 33.5 per 100,000 in 2000. During the study period, there were 3,020 children, who received a negative appendectomy. The negative appendectomy rates declined from 16.0 per 100,000 in 1993 to 10.2 per 100,000 in 2000, representing a 35.9% decrease.

### Geographic Variations of Appendectomy

Table 1 shows the age and sex-adjusted distribution of appendectomy rates by District Health Councils (DHCs) in Ontario in 1993 to 2000 sorted in ascending order from highest to lowest percentage of rural living. The overall age and sex-adjusted rates of positive, negative and perforated appendectomy were 93.2, 13.2 and 31.3 per 100,000 respectively. The detailed DHC-specific data showed that regions with higher percentage of rural living had higher positive, negative and perforated appendectomy rates with statistically significant correlation coefficients of 0.7, 0.6 and 0.6 respectively. For example, the Muskoka region (in Northern Ontario) which had 74% rural living had the highest rates in positive, negative and perforated appendectomy rates in the province. Their positive appendectomy and perforated appendectomy rates were more than 30% higher than the provincial average and their negative appendectomy rate was almost 2-fold that of the provincial rate.

**Table 1:** Sex-adjusted appendectomy rates per 100,000 children in the population less than nineteen years of age by district health council (DHC) of patient residence in Ontario, fiscal 1993-2000

District Health Council (DHC)	Percent Rural	Positive Appendectomy		Negative Appendectomy		Perforated Appendicitis	
		Number of Appendectomy Per Year †	Sex- Adjusted **Rate **‡	Number of Appendectomy Per Year †	Sex- Adjusted **Rate **‡	Number of Appendectomy Per Year †	Sex- Adjusted **Rate **‡
*sorted by ascending rank order * *of rural to urban)*							
Grey, Bruce, Huron, Perth	82	84.4	102.2	14.3	17.1	30.3	36.9
Muskoka, Nipissing, Parry Sound & Timiskaming	74	70.4	122.6*	15.0	26.0***	23.3	40.8
Quinte, Kingston, Rideau	39	133.0	107.8	15.6	12.3	15.6	29.5
Algoma, Cochrane, Manitoulin & Sudbury	37	115.9	98.4	16.1	13.6	36.9	31.8
Grand River	32	75.9	115.4	12.4	18.7	24.5	37.5
Northwestern Ontario	29	80.3	112.0	12.3	17.1	27.3	38.2
Waterloo Region-Wellington-Dufferin	23	186.8	106.0	32.4	18.4	65.8	37.3
Thames Valley	23	142.6	91.9	22.0	14.2	50.4	32.5
Essex, Kent, and Lambton	20	142.5	87.3	15.0	9.1	39.5	24.4
Durham, Haliburton, Kawartha & Pine Ridge	19	236.9	110.5**	47.1	22.2***	76.4	35.7
Champlain	16	262.0	100.5	24.8	9.5	78.6	30.1
Simcoe-York	9	243.6	88.3	32.4	11.7	93.4	33.7
Niagara Region	3	77.5	74.5*	12.8	12.3	24.4	23.6
Halton-Peel	0	283.3	83.2	38.0	11.1	95.6	28.0
Hamilton-Wentworth	0	96.6	80.4	8.3	6.8	38.0	31.6
Metropolitan Toronto	0	427.5	81.4**	59.1	11.3	152.0	28.9
							
Province-wide Age and Sex Adjusted Rate			93.20		13.19		31.29
							
***SMALL AREA VARIATION SUMMARY STATISTICS***							
Extremal Quotient [EQ]			1.65		3.80		1.73
Ratio of Third Quartile over First Quartile			1.28		1.59		1.27
Coefficient of Variation			13.09		32.04		12.95
Systematic Component of Variation			15.63		87.74		-0.10

† Average over time period

‡ Rate per 100,000

* p < 0.05; ** p < 0.01; *** p < 0.001

The p-values are based on the 1 degree of freedom chi-square test for the difference in appendectomy rates between each DHC and the province-wide rate.

The extremal quotients (EQ) in Table 1 represent the ratio of the highest to the lowest rates in Ontario, a measure of geographic variations. The EQs of positive and perforated appendectomies were 1.65 and 1.73 respectively indicated minimal variation of rates among geographic regions. As for negative appendectomy, as previously mentioned, Muskoka DHC had the highest rate (26.0 per 100,000) compared to the lowest rate of 6.8 per 100,000 in Hamilton-Wentworth DHC in Southern Ontario, representing approximately a 4-fold high to low ratio, a moderately large variation.

### Associations of Appendectomy and Population Demographic Factors

The relationships between appendectomy rates and socio-demographic variables were measured by the Spearman correlation coefficients (SCC). Rural living showed significant positive correlations with rates of primary appendectomy, negative appendectomy and perforated appendicitis (Table 2: SCC = 0.71, p < 0.01, SCC = 0.68, p < 0.01, SCC = 0.54, p < 0.05 respectively). On the other hand, the availability of surgeons in the DHCs showed negative correlations with appendectomy rates indicating that areas with more surgeons had lower negative appendectomy rates and lower perforated appendicitis (SCC = -0.74, p < 0.05 and SCC = -0.51, p < 0.05 respectively). Table 2 shows the level of rural living was negatively associated with ultrasound use (SCC = -0.30, p = 0.2565). The percentages of immigrants living in the DHCs were also negatively associated with negative appendectomy rates and perforated appendicitis, but the associations were not statistically significant.

**Table 2:** Association between socio-demographic variables and appendectomy and perforation rates

Variables		Pearson Correlation	
	Positive Appendectomy	Negative Appendectomy	Perforated Appendicitis
Rural living	0.71***	0.68**	0.54*
Availability of surgeon in the region	-0.69*	-0.74*	-0.51 *
Low family income	0.20	-0.09	-0.01
Home language: English	0.59*	0.59*	0.33
Below high school education	0.34	0.35	0.35
Ultrasound or CT use	-0.34	-0.12	-0.39
Percent Immigrants living in the region	-0.58*	-0.41	-0.39
	**Rural living**	**Percent Immigrants**	**Ultrasound/CT use**
Rural living	1.00	-0.52*	-0.30
Percent Immigrants living in the region	-0.52*	1.00	0.60*

* p < 0.05; ** p < 0.01; *** p < 0.001

### Logistic Regressions

Table 3 shows results from multivariable logistic regression analyses. Population demographic factors included in the analysis were rural living, percent immigrants living in the region, low family income, language used at home, percent of the population with at least high school education, the availability of surgeons in the region and the utilization rates of ultrasound were modeled against negative and perforated appendectomies. After adjusting for these factors, the odds of having a negative appendectomy were 28% higher in areas with rural living (OR = 1.28, 95% CI: 1.01, 1.61; p < 0.01). Areas with more available surgeons have a significantly lower odds of negative appendectomy (OR = 0.66, 95% CI: 0.52, 0.83; p < 0.01). Areas with higher ultrasound utilization had a significantly lower risk of perforated appendectomy (OR = 0.83, 95% CI: 0.72, 0.95; p < 0.05). Similarly, the odds of having a perforated appendectomy were higher in areas with higher level of rural living (OR = 1.11, 95% CI: 0.96, 1.28; p = 0.1682) after adjusting for other population demographic factors.

**Table 3:** Risk of negative or perforated appendectomy adjusted for confounders by logistic regression

Covariate	Negative Appendectomy		Perforated Appendicitis	
	OR† (95% CI)		OR† (95% CI)	
	Unadjusted	Adjusted	Unadjusted	Adjusted
Rural living	1.42 (1.16, 1.76)**	1.28 (1.01, 1.61)**	1.11 (0.96, 1.28)	
Availability of surgeon in the region	0.64 (0.52, 0.79)***	0.66 (0.52, 0.83)**	0.96 (0.83, 1.10)	
Low family income	0.99 (0.98, 1.00)	0.98 (0.97, 0.99)*	0.99 (0.99, 1.00)	0.99 (0.98, 0.99)*
Home language: English	1.01 (1.00, 1.02)		1.00 (0.99, 1.01)	
Below high school education §	1.01 (0.97, 1.06)*		1.00 (0.97, 1.03)	
Ultra-sound use	0.99 (0.95, 1.02)		0.98 (0.96, 1.01)	0.83 (0.72, 0.95)*
Immigrants	0.87 (0.69, 1.09)		1.01 (0.87, 1.18)	

† Odds Ratio and its corresponding 95% confidence intervals

§ Reference category is high school education

* p < 0.05, ** p < 0.001, *** p < 0.0001

## Discussion

Our study is the first to use population-based data to quantify the association of population demographic factors such as rural living and immigrant status with negative appendectomy and perforated appendicitis. We observed and reported significant and striking relationships between these factors and outcomes of appendectomy in children. Our results showed that rural living was significantly associated with higher odds of adverse outcomes of appendicitis (negative or perforated appendectomy). Furthermore, areas with a higher abdominal ultrasound use were associated with a lower risk of perforated appendectomy.

Recent studies have shown that the incidence of acute appendicitis vary substantially by geographic region, race, sex, immigrant status and socioeconomic status[1-4]. Researchers have hypothesized that environmental and genetic factors may account for some of the observed variations. A recent retrospective cohort study conducted by Smink et al[4] using inpatient data of children who had appendicitis in 22 states in the US showed that children uninsured or insured by Medicaid had a higher odds of perforation compared to those with private insurance indicating a disparity in access to care by socioeconomic status. However, in our study population, socioeconomic status of the child or the family did not affect the outcomes of appendectomy. This may be attributed to the fact that in Canada, children are universally covered by a public health insurance system for primary care visits, routine immunizations and medical or surgical hospitalizations; there may be fewer structural barriers to accessing health care.

The descriptive distributions of negative appendectomy rates by geographical regions in our Ontario data showed almost a 4-fold variation from high to low rates. In addition, the correlations of negative and perforated appendectomy are positively correlated with rural living, suggesting a higher negative and perforated appendectomy rates in areas with higher level of rural living. The higher risks of negative and perforated appendectomy in rural areas persisted after accounting for population demographic factors such as SES, the proportion of immigrants living in the region, use of English at home, low education, availability of surgeons in the residing region and the utilization of ultrasound. Our findings of higher negative and perforated appendectomy risks in rural areas suggested geographical distance to available health facilities, ultrasound use and the available of surgeons in the region could be significant factors that influence timely diagnosis and treatment of acute conditions such as appendicitis. Findings from other studies have also suggested that access disparities between rural and urban persist, even after adjusting for health insurance. There are often complex barriers to health care exist in rural areas that rural residents are less likely to receive regular check-ups and preventive screenings [19-21]. While abdominal pain in children is common, acute appendicitis diagnosed of children presented with acute abdominal pain is relatively low which increased the likelihood of an initial misdiagnosis[22-25]. Therefore, accurate evaluation of abdominal pain in children poses a major challenge. It is however extremely important to identify pain or symptoms associated with an acute appendicitis so as to prevent an adverse impact on the course of the disease such as a perforated appendix. The use and timely access to ultrasound may play a role in lowering the rate of negative appendectomy (appendectomy performed without a post-operative diagnosis of appendicitis) and the risk of a perforated appendicitis (where an accurate diagnosis of appendicitis was delayed).

Some recent studies have shown that patients of minority race are less likely to receive timely intervention for their conditions that may lead to less than desirable outcomes. For example, using the Pediatric Health Information System database Ponsky et al studied children who had appendectomies between 1997 and 2002 and showed that the rate of appendiceal rupture in school-aged children was associated with race and health insurance status, but not with negative appendectomy rate[3]. Similarly, the retrospective cohort study by Smink et al[4] suggested that perforated appendicitis disproportionately affected both children of minority race (black and Hispanic patients) and children insured by Medicaid. The findings suggested that black and Hispanic patients with acute appendicitis may present to medical attention later in their disease course. This inadequate access to medical care may contribute to their higher likelihood of developing appendicitis that was more prone to perforation. The study on Albanian immigrants in Greece by Tatsioni et al[1] also showed that Albanian immigrants had a higher risk for negative appendectomies. Their results suggested that cultural and language impediment may have been barriers to access to health care and therefore contributed to the poorer outcomes. Immigrants may be more likely exposed to gastrointestinal pathogens due to poor food and water hygiene that masquerade as appendicitis. In contrast to these published findings, our Canadian study showed that areas with higher percentage of immigrants were associated with lower positive, negative or perforated appendectomy rates. The higher negative appendectomy rates reported in the Albanian immigrant study could be attributed to cultural and language impediment that led to potential misdiagnosis. However, our findings of "immigrant status" being associated with a lower appendectomy rates or perforated appendicitis rate is in keeping with another Canadian study which showed a "healthy immigrant effect" among adults[26]. As well, a recent study conducted in three Canadian provinces showed that hospitalizations in immigrants were lower than Canadian-born residents and long-term immigrants between 1985 and 2000[27]. The lower rate of hospitalization may be partly explained by the good health status of immigrants, rather than poor access, highlighting the unique health patterns among them. However, it is still largely unknown whether immigrants to Canada are truly healthier, or whether they have different patterns of accessing health care or are treated differently by physicians making judgments about admissions. Other studies suggested dietary habit (e.g. higher level of consumption of dietary fibre) may play a role in the lower risk of appendicitis[28]. Ethnic origins and cultural practices may have an impact on some of the environmental influences thought to be related to appendicitis (such as high fiber diet). A future evaluation of the association of race/ethnicity with appendicitis would help rule out or clarify the impact of ethnicity on the rural/urban differences observed in our study.

## Conclusion

The higher rates of negative and perforated appendectomy in rural populations underpin the influence of access to preventive and primary health care in modifying the odds of appendicitis resulting in surgery.

## Competing interests

The authors declare that they have no competing interests.

## Authors' contributions

TTO designed and conducted the study, and was responsible for assembling the data cohort, supervising the statistical analyses, interpreting the findings and writing this manuscript. JCL provided clinical perspectives, critical revisions of the manuscript for important intellectual content. All authors read and approved the final manuscript.

## Pre-publication history

The pre-publication history for this paper can be accessed here:

http://www.biomedcentral.com/1472-6963/10/250/prepub
